# Anxiety and depression in pediatric patients with disorder of brain-gut interaction: the role of diarrhea and abdominal pain as key determinants

**DOI:** 10.3389/fped.2025.1628222

**Published:** 2025-08-26

**Authors:** Zhaodi Chen, Qiqi Chen, Li Zhou

**Affiliations:** ^1^ Department of Psychiatry, The Second Affiliated Hospital of Shandong First Medical University, Taishan, China; ^2^ Department of Pediatrics, The Second Affiliated Hospital of Shandong First Medical University, Taishan, China

**Keywords:** disorder of brain-gut interaction, pediatric patients, anxiety, depression, abdominal pain, diarrhea

## Abstract

**Objective:**

This study aimed to evaluate the prevalence of anxiety and depression in children diagnosed with disorder of brain-gut interaction (DGBI) and to examine their association with abdominal pain and diarrhea.

**Methods:**

This study employed a mixed-methods design, combining cross-sectional surveys with longitudinal follow-up, enrolling 311 children aged 6–18 years, including 119 in the DGBI group and 192 in the non-DGBI group. Psychological status was assessed using the Screen for Child Anxiety-Related Emotional Disorders (SCARED) and the Children's Depression Inventory-Second Edition (CDI-2). Symptom diaries were utilized to document abdominal pain intensity (measured via Visual Analog Scale, VAS) and diarrhea frequency. Multivariate logistic regression analysis was performed to identify significant risk factors.

**Results:**

The DGBI group exhibited significantly higher prevalence rates of anxiety (40.3% vs. 16.7%, *p* < 0.001) and depression (33.6% vs. 12.0%, *p* < 0.001) compared to the non-DGBI group. DGBI were associated with a 2.09-fold increased risk of anxiety (95% CI: 1.26–3.47) and a 3.09-fold increased risk of depression (95% CI: 1.76–5.45). The intensity and frequency of abdominal pain, as well as the frequency of diarrhea, were identified as independent predictors of both depression and anxiety. Notably, harmonious family relationships were found to significantly mitigate the risk of depression in children with DGBI.

**Conclusion:**

DGBI are strongly associated with elevated rates of anxiety and depression in pediatric populations, with abdominal pain and diarrhea serving as critical symptomatic drivers. Family support emerged as a protective factor against depression. These findings underscore the importance of routine psychological screening and the implementation of integrated, multidisciplinary interventions in the clinical management of DGBI.

## 1 Introduction

Disorder of brain-gut interaction (DGBI) are among the most common chronic gastrointestinal conditions affecting pediatric populations worldwide. These disorders are characterized by recurrent and often debilitating symptoms, including abdominal pain, diarrhea, constipation, bloating, and altered bowel habits, in the absence of detectable structural or biochemical abnormalities (1). DGBI represent a significant clinical challenge due to their high prevalence, chronic nature, and substantial impact on quality of life. According to the Rome IV diagnostic criteria, which provide a standardized framework for identifying functional gastrointestinal disorders, the median prevalence of DGBI is estimated to be 22.2% (range: 5.8%–40%) in children under 4 years of age and 21.8% (range: 19%–40%) in children aged 4–18 years (2). These figures highlight the widespread burden of DGBI across different pediatric age groups, making them a critical area of focus for both clinical practice and research. Among children over 4 years old, the most frequently diagnosed DGBI include functional constipation, functional dyspepsia, and irritable bowel syndrome (IBS) (3). Although DGBI are not life-threatening, they are often associated with significant psychological comorbidities, such as anxiety and depression, which markedly impair the quality of life in affected children and may persist into adulthood (4). The impact of DGBI on mental health has garnered increasing attention in recent years. Anxiety and depression are two of the most common psychiatric manifestations observed in children with DGBI (5). A meta-analysis demonstrated that children with DGBI exhibit significantly higher rates of anxiety and depressive symptoms compared to their healthy counterparts (6).

Emerging evidence suggests that specific DGBI symptoms, such as diarrhea and abdominal pain, may have distinct predictive value for psychological outcomes. Cross-sectional studies have shown that children with diarrhea-predominant IBS score 1.8 times higher on anxiety scales (e.g., SCARED) than those with constipation-predominant IBS, with elevated serum serotonin (5-HT) levels potentially implicating dysregulation in gut-brain monoamine neurotransmitter pathways (7). Furthermore, the duration and intensity of abdominal pain exhibit a dose-response relationship with depressive symptoms, as measured by the Children's Depression Inventory (CDI), suggesting that neural sensitization to pain may serve as a central mediator of emotional dysregulation (8). Functional abdominal pain (FAP) is intricately linked to heightened levels of anxiety and depressive symptoms, with a growing body of evidence underscoring its profound and enduring impact on mental health. Longitudinal studies have demonstrated that anxiety associated with FAP frequently originates in childhood and can persist well into late adolescence and early adulthood, even in cases where abdominal pain symptoms have resolved (9). This persistent psychological burden highlights the complex interplay between gastrointestinal dysfunction and emotional well-being, suggesting that FAP is not merely a transient somatic complaint but a condition with far-reaching implications for mental health.

This study aimed to evaluate the prevalence of anxiety and depression in pediatric DGBI patients compared to non-DGBI controls, while examining how specific gastrointestinal symptoms (abdominal pain and diarrhea) contribute to psychological distress. We further sought to identify protective factors like family relationships that might mitigate these mental health risks. Our findings provide crucial insights into the gut-brain axis in pediatric populations and highlight potential targets for integrated clinical interventions addressing both gastrointestinal and psychological symptoms in children with DGBI.

## 2 Methods

### 2.1 Study population

This study employed a mixed-methods design, combining cross-sectional surveys with longitudinal follow-up, to investigate the effects of diarrhea and abdominal pain on anxiety and depression in children with DGBI. Participants were recruited from pediatric outpatient and inpatient departments affiliated with our institution. Children diagnosed with DGBI were included, alongside a control group of healthy children undergoing psychological counseling during the same period, to evaluate whether DGBI and associated symptoms (diarrhea and abdominal pain) increase the risk of anxiety and depression.

All participants were minors (aged 6–18 years); thus, written informed consent was obtained from the parents or legal guardians of each participant, and assent was obtained from children as appropriate for their age and comprehension level. The study protocol was reviewed and approved by the institutional ethics committee.

### 2.2 Inclusion and exclusion criteria

#### 2.2.1 Inclusion criteria

For the DGBI group: (1) age 6–18 years; (2) meeting the Rome IV diagnostic criteria for functional abdominal pain (FAP) or IBS.

For the non-DGBI group: (1) age 6–18 years; (2) presenting to pediatric clinics for non-gastrointestinal issues, such as minor injuries, vaccinations, or routine health check-ups, with no history of gastrointestinal disorders (including DGBI) and no current gastrointestinal symptoms.

#### 2.2.2 Exclusion criteria

(1) Presence of severe organic diseases, including malignancies, significant cardiovascular disorders, or chronic kidney disease; (2) history of neurological conditions that may influence mental status or gastrointestinal function, such as epilepsy, cerebral palsy, or neurodegenerative diseases; (3) intellectual disabilities or developmental delays that preclude accurate completion of assessment tools; (4) current use of psychotropic medications, including antidepressants, anxiolytics, or stimulants, which may confound the assessment of anxiety and depressive symptoms; (5) language barriers that impede comprehension or completion of study questionnaires.

### 2.3 Measurement tools

#### 2.3.1 Anxiety and depression assessment

Anxiety was evaluated using the Child Anxiety-Related Emotional Disorders (SCARED) scale, a 41-item instrument (including 5 brief items) that assesses five domains: somatic/panic, generalized anxiety, separation anxiety, social phobia, and school phobia. The scale was completed by the child's parent or guardian using a 0–2 Likert scale, with higher scores indicating greater anxiety severity. A SCARED score ≥25 was defined as clinically significant anxiety (10).

Depression was assessed using the Children's Depression Inventory-Second Edition (CDI-2), a 27-item self-report measure that evaluates cognitive, affective, and behavioral symptoms of depression across five subscales: anhedonia, negative mood, low self-esteem, low efficacy, and interpersonal problems. Each item consists of three statements reflecting varying symptom severity (e.g., “I occasionally feel tired,” “I often feel tired,” “I always feel tired”), scored as 0, 1, or 2, yielding a total score of 54. Higher scores indicate more severe depressive symptoms. A CDI-2 score ≥19 was considered indicative of clinically significant depression (11).

#### 2.3.2 Gastrointestinal symptom assessment

Parents of children with DGBI were instructed to document the frequency and severity of abdominal pain and diarrhea over a one-week period. For abdominal pain, they recorded the location, intensity [using a visual analog scale ranging from 0 (no pain) to 10 (most severe pain imaginable)], duration, and frequency of episodes. For diarrhea, they recorded daily stool frequency, consistency (using the Bristol Stool Scale), and associated symptoms (e.g., urgency, incontinence).

### 2.4 Study enrollment and data collection

A total of 311 participants were initially enrolled, including 119 children with DGBI and 192 without DGBI. All participants completed the required assessments during the study period, and no participants withdrew consent or were lost to follow-up. All data were reviewed for completeness and consistency prior to analysis, and no data points were excluded due to implausibility or quality concerns.

Data collection was conducted by trained research assistants, including pediatric nurses and psychologists, who received comprehensive training on the administration of assessment tools, communication with participants and their families, and the importance of data accuracy.

Participants and their parents were briefed on the study objectives and procedures. Children completed the SCARED and CDI-2 questionnaires under the supervision of research assistants, while parents of DGBI patients were provided with symptom diaries and instructed on their proper use. Diaries were completed daily for two consecutive weeks and collected at the end of the study period.

### 2.5 Statistical analysis

Statistical analyses were performed using SPSS 27.0 and R 4.2.1. Descriptive statistics were computed for all variables, with continuous variables expressed as means ± standard deviations and categorical variables as frequencies and percentages. Group comparisons between DGBI and non-DGBI participants were conducted using chi-square tests for categorical variables (e.g., presence or absence of anxiety/depression). Multivariate logistic regression models were constructed to identify independent risk factors for anxiety and depression, with DGBI status (DGBI vs. non-DGBI), age, gender, and potential confounders (e.g., family history of mental illness, socioeconomic status) included as covariates. Odds ratios (OR) and 95% confidence intervals (CI) were calculated to quantify associations. A two-tailed significance level of *α* = 0.05 was applied for all analyses, except for multivariate logistic regression, where adjusted *p*-values were reported.

## 3 Results

### 3.1 Baseline characteristics of DGBI and non-DGBI groups

A total of 311 children were enrolled, including 119 with DGBI and 192 without DGBI. The demographic variables were well balanced, including gender, age, family income, parental education level, family history of mental illness, family relationships, residence, only-child status, or educational stage. However, children with DGBI exhibited significantly higher scores on both the SCARED and CDI-2 scales compared to the non-DGBI group (Table 1).

**Table 1:** Baseline characteristics of DGBI and Non-DGBI groups.

Characteristic	DGBI (*N* = 119)	Non-DGBI (*N* = 192)	Statistic	*p*-value
Age Group (years)			*χ*² = 0.66	0.717
6–11	41 (34.5%)	67 (34.9%)		
12–14	36 (30.3%)	65 (33.9%)		
15–18	42 (35.3%)	60 (31.3%)		
Sex			*χ*² = 0.20	0.657
Female	57 (47.9%)	87 (45.3%)		
Male	62 (52.1%)	105 (54.7%)		
Family income level			*χ*² = 0.59	0.743
Low	20 (16.8%)	38 (19.8%)		
Medium	61 (51.3%)	91 (47.4%)		
High	38 (31.9%)	63 (32.8%)		
Parental Education Level			*χ*² = 0.97	0.614
Primary	15 (12.6%)	30 (15.6%)		
Secondary	49 (41.2%)	83 (43.2%)		
Higher	55 (46.2%)	79 (41.1%)		
Family history of mental illness			*χ*² = 0.50	0.480
No	105 (88.2%)	164 (85.4%)		
Yes	14 (11.8%)	28 (14.6%)		
Family Relationship Quality			*χ*² = 1.17	0.557
Poor	8 (6.7%)	15 (7.8%)		
Fair	23 (19.3%)	46 (24.0%)		
Good	88 (73.9%)	131 (68.2%)		
Residence			*χ*² = 0.93	0.334
Rural	48 (40.3%)	67 (34.9%)		
Urban	71 (59.7%)	125 (65.1%)		
Only child			*χ*² = 0.37	0.543
No	69 (58.0%)	118 (61.5%)		
Yes	50 (42.0%)	74 (38.5%)		
SCARED score			W = 13,008.00	0.040
Mean ± SD	22 ± 9	20 ± 6		
Median (IQR)	22 (17, 28)	20 (16, 25)		
Range	1–49	2–36		
CDI 2 score			W = 13,270.00	0.017
Mean ± SD	16.3 ± 5.9	14.3 ± 4.4		
Median (IQR)	15.4 (11.8, 21.3)	14.4 (11.6, 16.9)		
Range	3.8–33.7	2.5–25.3		

Age groups were defined to reflect typical educational stages and psychological development phases: elementary (6–11), early adolescence (12–14), and late adolescence (15–18).

Family income categories were defined based on gross monthly household income: Low (<¥5,000), Medium (¥5,000–10,000), High (>¥10,000).

Parental education was categorized as: Primary (elementary school or below), Secondary (middle/high school), and Higher (college/university or above).

Family relationship quality was self-reported or rated by parents as: Poor (frequent conflicts, low support), Fair (occasional conflicts, moderate support), Good (harmonious, supportive relationships).

### 3.2 Risk factor analysis for depression and anxiety

Using a SCARED score of ≥25 as the cutoff for clinically significant anxiety and a CDI-2 score of ≥19 as the cutoff for clinically significant depression, multivariate logistic regression analysis was performed with the presence or absence of anxiety and depression as the dependent variables. Among the study participants, 67 cases of depression were identified, of which 40 were in the DGBI group, and 96 cases of anxiety were identified, of which 48 were in the DGBI group. The prevalence of depression and anxiety was markedly elevated in the DGBI group relative to the non-DGBI group, a finding that underscores the profound psychological burden associated with functional gastrointestinal disorders in pediatric populations. To elucidate the factors contributing to this increased risk, both univariate and multivariate logistic regression analyses were systematically conducted. The results demonstrated that harmonious family relationships served as protective factors against both depression and anxiety, while older age (16–18 years) was a protective factor against anxiety. Conversely, DGBI were identified as a significant risk factor for both depression and anxiety (Table 2).

**Table 2:** Univariate and multivariate logistic regression analysis of risk factors for depression and anxiety.

Characteristic	Depression univariable OR	Depression univariable 95% CI	Depression univariable *p*-value	Depression multivariable OR	Depression multivariable 95% CI	Depression multivariable *p*-value	Anxiety univariable OR	Anxiety univariable 95% CI	Anxiety univariable *p*-value	Anxiety multivariable OR	Anxiety multivariable 95% CI	Anxiety multivariable *p*-value
Age: 6–11	–	–	–	–	–	–	–	–	–	–	–	–
Age: 12–14	1.38	0.70, 2.72	0.351	1.38			0.66	0.37, 1.18	0.164	0.72	0.40, 1.32	0.288
Age: 15–18	1.52	0.78, 2.97	0.220	1.52			0.48	0.27, 0.88	0.017	0.50	0.27, 0.94	0.030
Sex: Female	–	–	–	–	–	–	–	–	–	–	–	–
Sex: Male	1.17	0.68, 2.01	0.576				0.63	0.39, 1.03	0.064	0.61	0.37, 1.01	0.053
Family Income: Low	–	–	–	–	–	–	–	–	–	–	–	–
Family Income: Medium	0.57	0.28, 1.14	0.112				0.90	0.48, 1.71	0.757			
Family Income: High	0.63	0.30, 1.33	0.227				0.69	0.34, 1.39	0.304			
Parental Education: Primary	–	–	–	–	–	–	–	–	–	–	–	–
Parental Education: Secondary	0.69	0.31, 1.55	0.364				1.25	0.57, 2.72	0.574			
Parental Education: Higher	0.97	0.44, 2.13	0.939				1.67	0.78, 3.60	0.190			
Family History Mental Illness: No	–	–	–	–	–	–	–	–	–	–	–	–
Family History Mental Illness: Yes	0.70	0.29, 1.65	0.411				1.29	0.65, 2.55	0.466			
Family Relationship: Poor	–	–	–	–	–	–	–	–	–	–	–	–
Family Relationship: Fair	0.55	0.22, 1.38	0.206	0.49	0.19, 1.27	0.144	0.50	0.21, 1.19	0.119	0.49	0.20, 1.19	0.115
Family Relationship: Good	0.28	0.09, 0.85	0.025	0.27	0.09, 0.85	0.025	0.33	0.12, 0.89	0.028	0.32	0.11, 0.90	0.031
Residence: Rural	–	–	–	–	–	–	–	–	–	–	–	–
Residence: Urban	1.07	0.61, 1.87	0.825				1.03	0.63, 1.70	0.899			
Only Child: No	–	–	–	–	–	–	–	–	–	–	–	–
Only Child: Yes	1.40	0.81, 2.41	0.228				1.34	0.82, 2.19	0.237			
DGBI: Non-DGBI	–	–	–	–	–	–	–	–	–	–	–	–
DGBI: DGBI	3.09	1.77, 5.40	<0.001	3.09	1.76, 5.45	<0.001	2.03	1.24, 3.31	0.005	2.09	1.26, 3.47	0.004

### 3.3 Relationship between gastrointestinal symptoms and anxiety/depression in DGBI patients

Abdominal pain and diarrhea represent the hallmark symptoms of DGBI. For depression, multivariate logistic regression analysis revealed that the severity and frequency of abdominal pain, the frequency of diarrhea, and harmonious family relationships were significant predictors of depression in DGBI patients. Specifically, more frequent and severe abdominal pain, as well as more frequent diarrhea, were associated with elevated levels of depression in children with DGBI. In contrast, harmonious family relationships were associated with reduced levels of depression (Table 3).

**Table 3:** Multivariate logistic regression analysis of factors influencing depression in DGBI patients.

Characteristic	Univariable OR	Univariable 95% CI	Univariable *p*-value	Multivariable OR	Multivariable 95% CI	Multivariable *p*-value
Age: 6–11	—	—	—	—	—	—
Age: 12–14	1.08	0.42, 2.77	0.867			
Age: 15–18	1.73	0.67, 4.44	0.257			
Sex: Female	—	—	—	—	—	—
Sex: Male	1.19	0.56, 2.56	0.653			
Family income: Low	—	—	—	—	—	—
Family income: Medium	0.43	0.15, 1.20	0.107	0.58	0.15, 2.18	0.416
Family income: High	0.22	0.07, 0.71	0.011	0.20	0.04, 0.92	0.039
Parental education: Primary	—	—	—	—	—	—
Parental education: Secondary	0.88	0.26, 3.03	0.842			
Parental education: Higher	1.14	0.34, 3.82	0.828			
Family history mental illness: No	—	—	—	—	—	—
Family history mental illness: Yes	1.11	0.35, 3.57	0.859			
Family relationship: poor	—	—	—	—	—	—
Family relationship: fair	0.73	0.14, 3.93	0.713			
Family relationship: good	0.86	0.19, 3.85	0.846			
Residence: Rural	—	—	—	—	—	—
Residence: Urban	0.87	0.40, 1.89	0.732			
Only child: No	—	—	—	—	—	—
Only child: Yes	1.91	0.88, 4.12	0.101			
Abdominal pain VAS score	1.47	1.19, 1.82	<0.001	1.32	1.03, 1.70	0.027
Abdominal pain frequency	17.82	1.94, 77.35	<0.001	15.21	11.82, 47.42	0.003
Diarrhea frequency	11.90	2.26, 62.87	0.002	12.12	2.27, 73.53	0.004
Vomiting frequency	17.48	1.23, 85.02	0.004	19.52	0.82, 69.28	0.071

For anxiety, multivariate logistic regression analysis indicated that the severity and frequency of abdominal pain and the frequency of diarrhea were significant predictors of anxiety in DGBI patients. More frequent and severe abdominal pain, as well as more frequent diarrhea, were associated with higher levels of anxiety in children with DGBI (Table 4).

**Table 4:** Multivariate logistic regression analysis of factors influencing anxiety in DGBI patients.

Characteristic	Univariable OR	Univariable 95% CI	Univariable *p*-value	Multivariable OR	Multivariable 95% CI	Multivariable *p*-value
Age: 6–11	—	—	—	—	—	—
Age: 12–14	0.35	0.14, 0.86	0.022	0.46	0.10, 2.19	0.329
Age: 15–18	0.55	0.22, 1.36	0.197	0.47	0.08, 2.73	0.400
Sex: Female	—	—	—	—	—	—
Sex: Male	0.37	0.17, 0.78	0.010	0.40	0.10, 1.61	0.197
Family income: Low	—	—	—	—	—	—
Family income: Medium	1.11	0.40, 3.12	0.837			
Family income: High	0.88	0.29, 2.66	0.814			
Parental education: Primary	—	—	—	—	—	—
Parental education: Secondary	1.50	0.45, 5.05	0.513			
Parental education: Higher	1.33	0.40, 4.43	0.639			
Family history mental illness: No	—	—	—	—	—	—
Family history mental illness: Yes	1.56	0.51, 4.78	0.435			
Family relationship: Poor	—	—	—	—	—	—
Family relationship: Fair	0.89	0.17, 4.72	0.890			
Family relationship: Good	1.21	0.27, 5.38	0.803			
Residence: Rural	—	—	—	—	—	—
Residence: Urban	0.79	0.37, 1.66	0.533			
Only child: No	—	—	—	—	—	—
Only child: Yes	0.85	0.40, 1.78	0.658			
Abdominal pain VAS Score	1.70	1.36, 2.12	<0.001	1.50	1.07, 2.10	0.020
Abdominal pain frequency	67.65	4.04, 973.65	<0.001	11.95	1.56, 75.10	0.003
Diarrhea frequency	12.82	5.76, 41.53	<0.001	13.04	4.34, 68.88	0.008
Vomiting frequency	22.51	1.50, 87.80	<0.001	13.7	0.85, 37.69	0.136

## 4 Discussion

This study, leveraging a robust sample size (*n* = 311), provides compelling evidence for the significant association between DGBI and the heightened prevalence of anxiety and depression in pediatric populations. The DGBI group exhibited markedly higher rates of anxiety (40.3%) and depression (33.6%) compared to the non-DGBI group (16.7% and 12.0%, respectively; *p* < 0.001). These findings are consistent with prior longitudinal research, such as the study by Shelby et al., which demonstrated a 2.3-fold increased risk of anxiety disorders in adulthood among individuals with a history of childhood functional abdominal pain (9). The results underscore the enduring psychological burden associated with DGBI, extending beyond gastrointestinal symptoms to encompass significant mental health comorbidities.

Multivariate logistic regression analysis further identified DGBI as an independent risk factor for both anxiety (OR = 2.09, 95% CI: 1.26–3.47) and depression (OR = 3.09, 95% CI: 1.76–5.45). These findings align with the work of Lee et al., who posited that FGID patients are more susceptible to depression, severe anxiety, and childhood trauma, all of which may exacerbate or perpetuate gastrointestinal symptoms (12). Notably, the elevated SCARED and CDI-2 scores observed in the DGBI group suggest a bidirectional relationship between gastrointestinal dysfunction and psychological distress. This phenomenon may be mediated by dysregulation of the gut-brain axis, wherein gut microbiota dysbiosis influences the limbic system via vagal pathways, leading to imbalances in key neurotransmitters such as serotonin and gamma-aminobutyric acid (GABA) (13). Such neurochemical disruptions are increasingly recognized as central to the pathophysiology of both DGBI and mood disorders.

A novel contribution of this study is the identification of abdominal pain intensity and diarrhea frequency as independent predictors of depression in children with DGBI. These findings corroborate the clinical intervention study by Youssef et al., which reported a 68% improvement in depressive symptoms following cognitive behavioral therapy (CBT) aimed at reducing the frequency of abdominal pain (14). Mechanistically, chronic abdominal pain may contribute to mood dysregulation through multiple pathways: (1) neural sensitization, wherein persistent nociceptive input enhances the excitability of dorsal horn neurons, leading to central sensitization and amplified pain perception (15, 16); (2) activation of the hypothalamic-pituitary-adrenal (HPA) axis, resulting in elevated cortisol levels and heightened stress responses (17); and (3) impaired social functioning, as frequent pain episodes increase school absenteeism and contribute to social withdrawal (18). These pathways collectively underscore the complex interplay between somatic symptoms and psychological well-being in pediatric DGBI.

The impact of diarrhea on emotional health was equally pronounced, with each additional daily episode increasing the risk of anxiety by 13.04-fold. This association may be attributed to the profound sense of loss of control and social stigma associated with diarrhea, as well as its disruptive effects on daily activities. Saps et al. found that 62% of children with diarrhea-predominant DGBI exhibited social avoidance behaviors, further highlighting the psychosocial burden of this symptom (19). Additionally, diarrhea-related gut microbiota dysbiosis may directly influence mood through the gut-brain axis. Emerging evidence suggests that specific probiotic strains, such as Lactobacillus and Bifidobacterium, can modulate GABA receptor activity and serotonin synthesis, thereby ameliorating anxiety-like behaviors (20). These findings underscore the potential for microbiota-targeted interventions in the management of DGBI and associated mood disorders.

Family relationships emerged as a critical protective factor in this study, with good family relationships, as defined by high levels of emotional support and low conflict, associated with a 73% reduced risk of depression (OR = 0.27, *p* = 0.025). These responses—coded as “good” on a standardized 3-point family relationship scale—reflect what we refer to as harmonious family dynamics, a term used here to denote emotionally supportive and cohesive household environments. This finding aligns with the seminal work of Walker et al., which emphasized the role of parental reinforcement in shaping illness behaviors and emotional responses to abdominal symptoms (21). Supportive family environments may mitigate psychological distress through several mechanisms: (1) emotional buffering, wherein parental attentiveness and empathy reduce stress and foster resilience (22); (2) enhanced treatment adherence, as family involvement has been shown to increase medication compliance by up to 40% (23); and (3) behavioral modeling, wherein positive coping strategies are reinforced through family interactions. Interestingly, family income was inversely correlated with depression (OR = 0.20, *p* = 0.039), likely reflecting greater access to healthcare resources and psychological support in higher-income households (24). However, parental education level did not significantly influence outcomes, consistent with findings by Hyams et al., which suggest that emotional support within the family unit may outweigh socioeconomic status in its impact on mental health outcomes (25).

These findings have important implications for the comprehensive management of DGBI. Routine screening for anxiety and depression using validated tools such as the SCARED and CDI-2 is recommended for all children with DGBI (American Academy of Pediatrics Grade B recommendation). In addition to conventional gastrointestinal treatments, integrative approaches such as CBT and family-based interventions should be incorporated into clinical practice, as endorsed by NICE guidelines. Future research should explore the molecular mechanisms underlying the gut-brain axis, with a particular focus on serotonin transporter gene polymorphisms (e.g., SLC6A4) and their role in mediating symptom-emotion interactions.

Despite its contributions, this study has several limitations. The cross-sectional design precludes causal inferences, and reliance on parent-reported symptom diaries may introduce measurement bias. Additionally, the sample was drawn from a single geographic region, limiting the generalizability of the findings. Future studies should employ longitudinal designs with follow-up periods of at least five years, incorporate objective biomarkers (e.g., fecal cortisol and microbiota profiling), and validate findings across diverse cultural and socioeconomic contexts. Promising research directions include investigating the efficacy of mindfulness-based stress reduction (MBSR) in pediatric DGBI, elucidating the role of the gut microbiota-gut-brain axis in mood regulation, and exploring the clinical applications of virtual reality (VR) for pain management.

## 5 Conclusion

This large-scale study provides robust evidence for the significant association between DGBI and anxiety/depression in children, identifying abdominal pain intensity and diarrhea frequency as key predictors of mood disorders. Family support emerged as a critical protective factor, highlighting the importance of a biopsychosocial approach to DGBI management. The findings underscore the need for routine psychological screening and integrative interventions in clinical practice. Future research should focus on longitudinal studies to establish causality, elucidate the molecular mechanisms of the gut-brain axis, and evaluate the long-term efficacy of family-based and microbiota-targeted therapies.

## Data Availability

The original contributions presented in the study are included in the article/Supplementary Material, further inquiries can be directed to the corresponding author.
